# AtPGL3 is an Arabidopsis BURP domain protein that is localized to the cell wall and promotes cell enlargement

**DOI:** 10.3389/fpls.2015.00412

**Published:** 2015-06-09

**Authors:** Jiyoung Park, Yong Cui, Byung-Ho Kang

**Affiliations:** ^1^Plant Molecular Cellular Biology Program, Microbiology and Cell Sciences, University of FloridaGainesville, FL, USA; ^2^State Key Laboratory of Agrobiotechnology, School of Life Sciences, The Chinese University of Hong KongHong Kong, China

**Keywords:** BURP domain protein, cell expansion, cell wall protein, alpha-expansin, polygalacturonase beta subunit

## Abstract

The BURP domain is a plant-specific domain that has been identified in secretory proteins, and some of these are involved in cell wall modification. The tomato polygalacturonase I complex involved in pectin degradation in ripening fruits has a non-catalytic subunit that has a BURP domain. This protein is called polygalacturonase 1 beta (PG1β) and the *Arabidopsis* genome encodes *three* proteins that exhibit strong amino acid similarities with PG1β? We generated Arabidopsis lines in which expression levels of *AtPGLs* are altered in order to investigate the biological roles of the Arabidopsis PG1β-like proteins (AtPGLs). Among the three *AtPGLs* (*AtPGL1-3*), *AtPGL3* exhibited the highest transcriptional activity throughout all developmental stages. *AtPGL* triple mutant plants have smaller rosette leaves than those of wild type plants because the leaf cells are smaller in the mutant plants. Interestingly, when we overexpressed *AtPGL3* using a 35S promoter, leaf cells in transgenic plants grew larger than those of the wild type. A C-terminal GFP fusion protein of AtPGL3 complemented phenotypes of the triple mutant plants and it localized to the cell wall. A truncated AtPGL3-GFP fusion protein lacking the BURP domain failed to rescue the mutant phenotypes even though the GFP protein was targeted to the cell wall, indicating that the BURP domain is required for the protein's effect on cell expansion. Quantitative RT-PCR and immunoblot analyses indicated that the α-expansin 6 gene is up-regulated in the overexpressor plants. Taken together, these results indicate that AtPGL3 is an apoplastic BURP domain protein playing a role in cell expansion.

## Introduction

Plant cells are enclosed within sturdy cell walls that make them tolerant of turgor pressure (Baskin, 2005). However, in growing plant cells, the cell wall is loosened to increase its extensibility, and turgor pressure stretches the loosened cell wall until the turgor pressure and tensile strength of the cell wall counterbalance each other. The control of wall extensibility determines cell sizes in plants and plays a critical role in the development of plant organs (Boudaoud, 2010).

The plant cell wall is composed of polysaccharides, proteins, and phenolic compounds (Levy and Staehelin, 1992). Cellulose microfibrils are paracrystalline fibers which are primarily responsible for the tensile strength of the cell wall. The cellulose fibers are embedded in a matrix consisting of hemicellulose and pectin (Somerville et al., 2004). Phenolic compounds are abundant in the secondary cell wall that is deposited inside the primary cell wall after cell expansion is completed. Structural proteins, as well as proteins which play roles in defense, environmental sensing, and intercellular signaling, are found in the cell wall (Nuhse, 2012). The plant cell wall is a dynamic compartment in which the composition and organization of its constituents are modified according to developmental programs or in response to environmental cues. Cell expansion in plants accompanies cell wall modification (Marga et al., 2005) and proteins involved in cell wall loosening have been shown to contribute to cell expansion.

One of the best-known cell wall proteins involved in cell expansion is the expansin family of proteins. They constitute a family of ~30 kDa proteins that are thought to be the primary promoters of cell extensibility. They do not have enzymatic activities, but they are capable of loosening the wall rapidly in a pH-dependent manner, although their exact mechanism is not understood (Cosgrove, 2005). Proteins with enzymatic activities that directly modify cell wall polysaccharides have also been shown to influence wall extensibility. Deesterification of pectin polysaccharides by methylesterases (Pelletier et al., 2010) or by acetylesterase frees carboxylic groups from the polysaccharides (Gou et al., 2012). Crosslinking of pectin polysaccharides by Ca^2+^ stiffens the cell wall and thereby inhibits cell elongation. Recently a pectin-digesting enzyme termed polygalacturonase involved in the cell expansion1 (PGX1) was shown to promote cell expansion in hypocotyls (Xiao et al., 2014). It was suggested that PGX1 loosens the cell wall by directly cleaving homogalacturonan.

The BURP domain is a plant-specific protein domain characterized by a highly conserved amino acid sequence motif (Xu et al., 2010). After its first identification in a storage protein of *Brassica napus*, termed BNM2, the domain has been detected in many secretory proteins of monocotyledonous and dicotyledonous plants. The name BURP is an acronym derived from the four representative proteins containing the domain, which are **B**NM2, **U**SP, **R**D22, and **P**G1β (Hattori et al., 1998). BNM2 and USP are storage proteins that are targeted to protein storage vacuoles (Van Son et al., 2009), while RD22 and PG1β are deposited in the cell wall (Wang et al., 2012). These two apoplastic BURP domain proteins have been implicated in cell wall relaxation. GhRDL1 is a cotton RD22 family protein that is highly expressed in elongating fiber cells. This protein promotes the enlargement of fiber cells when over-expressed. Because GhRDL1 interacts directly with a cotton α-expansin, it is thought that expansins mediate GhRDL1's effect on cell enlargement (Xu et al., 2013).

PG1β is a subunit of the tomato PG1 complex that dissolves pectin polysaccharides in the cell walls of tomato fruits when they ripen (Watson et al., 1994). The protein complex consists of two subunits. PG2 is the catalytic subunit that cleaves pectin polysaccharides and PG1β is the non-catalytic subunit (Dellapenna et al., 1990). However, comprehensive expression analyses of BURP domain-carrying genes of rice and soybean (Ding et al., 2009; Xu et al., 2010) have indicated that transcription of PG1β-like genes occurs in almost all plant tissues, and that the expression of several PG1β-like genes altered with changes in growth conditions, suggesting that PG1β-like proteins have functions other than pectin degradation during fruit softening.

In this study, we isolated T-DNA mutants of Arabidopsis PG1β-like genes (*AtPGLs*) and generated overexpressor lines of *AtPGL3* to examine the functions of *AtPGLs*. The Arabidopsis genome contains three genes encoding PG1β-like proteins (AtPGL1, AtPGL2, and AtPGL3) and triple mutant lines of the genes displayed a reduction in rosette leaf sizes, while rosette leaves of an *AtPGL3* overexpressor line were larger than those of the wild type. We also demonstrated that the BURP domain is required for the normal function of AtPGL3, and that expression levels of an α-expansin are related to those of *AtPGL3* in the transgenic lines.

## Results

### The arabidopsis polygalacturonase 1β subunit-like proteins, AtPGL family

**Figure 1:**
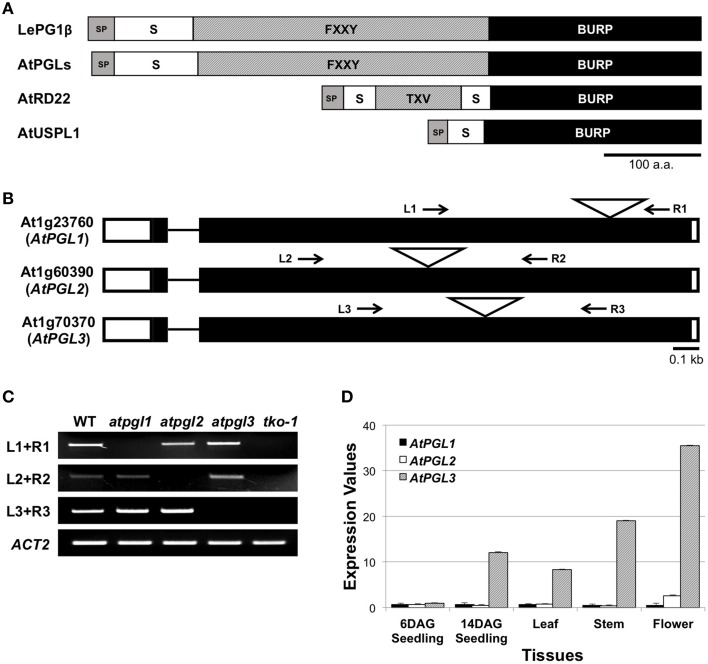
**BURP domain proteins of** ***Arabidopsis***. **(A)** Domain structures of the tomato polygalacturonase 1β (LePG1β) and three classes of Arabidopsis BURP domain proteins. SP, signal peptide; S, short segment; TXV, TXV repeats where T, V, and X stand for threonine, valine, and any amino acid, respectively. FXXY, FXXY repeats where F, Y, and X stand for phenylalanine, tyrosine, and any amino acid, respectively. Scale bar = 100 amino acids. **(B)** Exon-intron structures of the three *AtPGL* genes. They have two exons (rectangles) connected by a short intron (line). Positions of T-DNA insertion are marked with triangles. White boxes at each end of the gene represent 5′- and 3′-UTR. Arrows indicate primer binding sites for genotyping and for RT-PCR analyses in **(C)**. **(C)** Transcripts from *AtPGL* genes are amplified in the WT, the three single mutants (*atpgl1, atpgl2, and atpgl3*), and the *atpgl1;2;3* triple mutant (*tko-1*). *ACT2* (*actin2*) is utilized as a control. **(D)** Expression levels of the three *AtPGLs* in different tissues measured by qRT-PCR. Error bars represent standard deviations of the mean values.

We identified three open reading frames in the Arabidopsis genome that encode proteins with significant amino acid sequence identity (45.7%) to the tomato polygalacturonase1β subunit (LePG1β) (Figure S1). These Arabidopsis proteins and LePG1β share similar domain architectures, with signal peptides at the N-terminus followed by short segment repeats, FXXY (where F is phenylalanine, Y is tyrosine, and X is any amino acid), and with BURP domains at the C-terminus (Figure 1A). We named these three Arabidopsis LePG1β-like proteins, AtPGL1, AtPGL2, and AtPGL3. They share approximately 59% amino acid identity with each other (Figure S2).

### Expression patterns of AtPGL family genes

We performed quantitative reverse transcription PCR (qRT-PCR) to determine transcript levels of the three *AtPGL* genes. We first verified the specificity of each primer set using semi-qRT-PCR of total RNA samples from T-DNA-inserted mutant lines of the three genes (Figures 1B,C). *AtPGLs* are highly expressed in flowers and stems in mature Arabidopsis plants. *AtPGL* transcript levels were measured high in seedlings at 14 days after germination (DAG) but were barely detectable in six DAG seedlings. Transcriptional activities of *AtPGL1* and *AtPGL2* were 10–20 times lower than those of *AtPGL3* in all tissues that we examined, indicating that *AtPGL3* is the most highly transcribed member of the family (Figure 1D). Expression profiles of the *AtPGL* genes obtained from the Genevestigator database (Hruz et al., 2008) were consistent with our qRT-PCR results (Figure S3).

To determine cell- and tissue-specific expression patterns of *AtPGL3*, we generated transgenic lines containing an *AtPGL3* promoter (1.7 kbp from the start codon) plus bacterial uidA β-glucuronidase (GUS) translational fusion construct, AtPGL3-GUS. The *AtPGL3* promoter utilized in the ADL1C- GUS reporter construct was sufficient to control the expression of AtPGL3 for molecular complementation of the *atpgl* triple knockout mutant (*tko-1;* see below). Eight *AtPGL3-GUS* transformed lines were examine to determine tissue-specific promoter activities by GUS staining.

**Figure 2:**
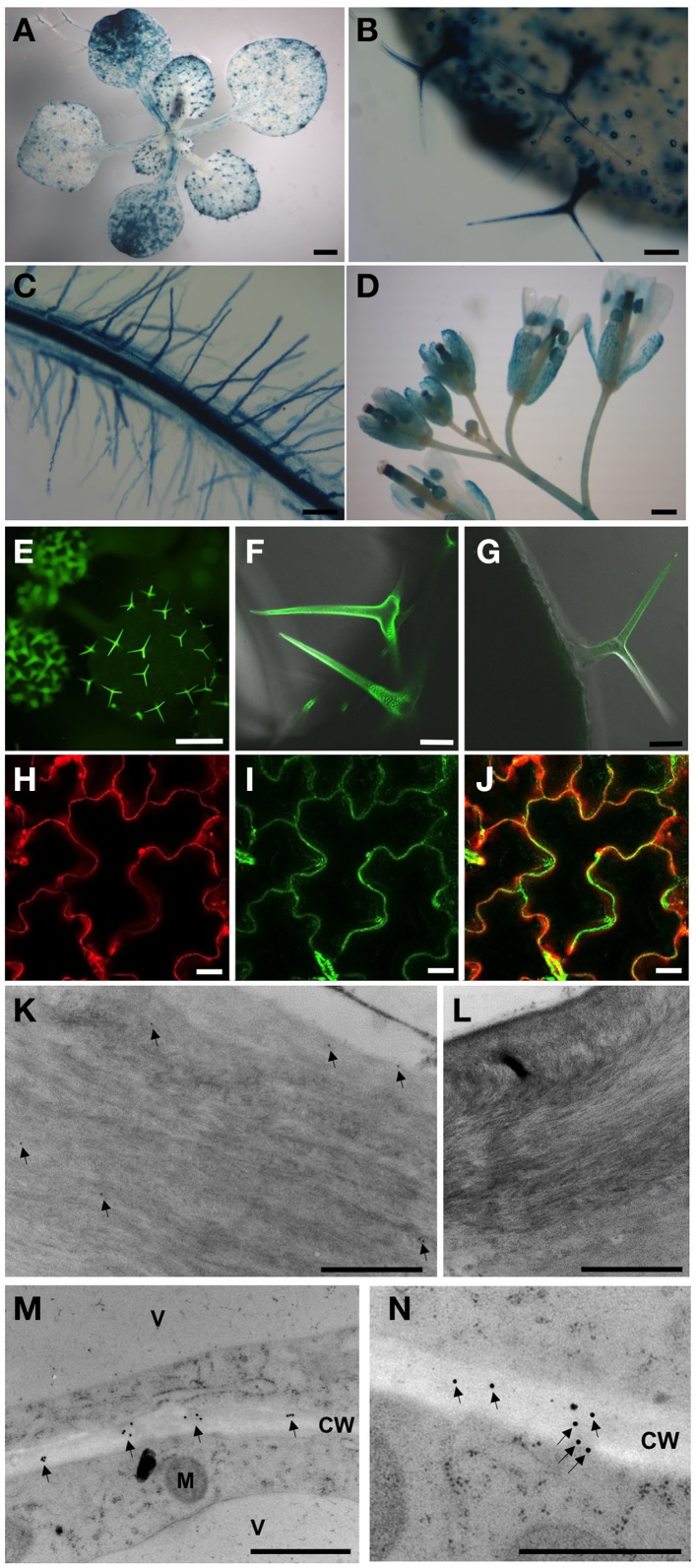
**Activities of the** ***AtPGL3*** **promoter (A–D) and localization of AtPGL3-GFP (E–N). (A)** 18-day-old seedling. **(B,C)** Trichomes **(B)**, guard cells **(B)**, epidermal cells **(B)** and root **(C)** of 18-day-old plants. **(D)** Flowers. **(E–J)** AtPGL3-GFP concentrates to trichomes **(E–G)** and the epidermal cell wall **(H,J)**. Trichome and epidermal cell images were captured from 18 day-old and 28-day-old plant leaves, respectively. To visualize the cell wall, leaves were stained with propidium iodide **(H)** and that image was merged with **(I,J)**. **(K–N)** Immunogold labeling of AtPGL3-GFP with a GFP antibody. GFP-specific immunogold particles were detected in trichome cell walls (arrows in **K**) and epidermal cell walls (arrows in **M**) from 21-day-old plant leaves. **(N)** Higher magnification of AtPGL3-GFP. No gold particles were observed in trichome cell walls **(L)** or epidermal cell walls (Figure S4) of wild type seedlings. Scale bars in **(A,D**,**E)** = 0.1 cm. Scale bars in **(B,C)** = 0.1 mm. Scale bars in **(F,G)** = 50 μm. Scale bars in **(H–J)** = 20 μm. Scale bars in **(K–N)** = 1 μm.

*AtPGL3*-*GUS* activity was detected in trichomes and guard cells in seedling leaves. *AtPGL3*-*GUS* staining was not detected in the shoot apical meristem, but weak staining was observed in expanding leaves (Figures 2A,B). The vascular tissue in the leaves did not exhibit any GUS staining but vascular tissue in the root was strongly stained. Epidermal cells of roots, including the root hairs, were positive for GUS staining, analogous to the trichomes and guard cells of the aerial tissues (Figure 2C). Pollen sacs, sepals, and styles of pistils exhibited *AtPGL3*-*GUS* activity (Figure 2D), consistent with the qRT-PCR results (Figure 1D).

### Defective cell expansion in atpgl1; atpgl2; atpgl3 triple knockout mutant (tko-1) seedling leaves

**Figure 3:**
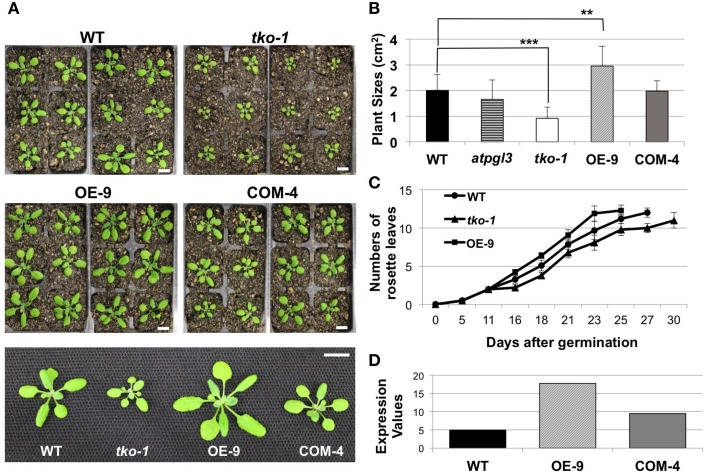
**Changes in plant sizes. (A)** 21-day-old wild type (WT), *tko-1*, overexpressor (OE-9), and complemented line (COM-4) plants. Scale bars = 1 cm. **(B)** Average plant sizes. Error bars represent standard deviation. The significance of size differences between WT, *tko-1*, OE-9, and COM-4 was determined by Student's *t*-test. ^**^P < 0.01, ^***^P < 0.001 (*n* = 22, 17, 39, and 33 for WT, *tko-1*, OE-9, and COM-4, respectively). **(C)** Average numbers of rosette leaves at days after germination (*n* = 22, 17, and 21 for WT, *tko-1*, and OE-9, respectively). **(D)** Expression values of *AtPGL3* derived from gel intensity measured from semi-quantitative RT-PCR in the overexpressor line (OE-9) and the complemented line (COM-4) shown in **(A–C)**, compared to WT.

To better understand the role(s) of *AtPGLs* in Arabidopsis development, we generated Arabidopsis mutant plants in which all three *AtPGL* genes were disrupted by T-DNA insertions. We acquired mutant lines of each *AtPGL* gene from the Salk T-DNA collections. The T-DNA lines were backcrossed to the wild type (WT), Col-0, three times and homozygous lines of *atpgl1, atpgl2*, and *atpgl3* were isolated. Homozygous *atpgl1* and homozygous *atpgl2* mutant lines did not exhibit any phenotypic defect throughout their life cycle. The *atpgl1; atpgl2* double homozygous mutant plants were also indistinguishable from WT plants. To generate triple mutant lines in which all three *AtPGLs* were inactivated, we crossed the double homozygous line to the *atpgl3* single mutant plants. Inactivation of all three *AtPGL* genes was verified in triple mutant plants (*tko-1*) by PCR genotypic analysis and by RT-PCR (Figures 1B,C). The triple mutant plants produced leaves and petioles that were smaller than those of WT seedlings when germinated on soil and grown side by side (Figures 3A,B). Sizes of *atpgl3* single homozygous plants were slightly reduced when compared with sizes of WT plants (Figure 3B). Disruption of functional copies of *AtPGL1* and *AtPGL2* amplified the size reduction phenotype, suggesting that the functions of these genes overlap (Figures 3A,B). It was observed that the *tko-1* mutant plants produced less numbers of rosette leaves than wild type plants did from ~11 days after germination. Around 28–30 days after germination, similar numbers of rosette leaves were seen in *tko-1* mutant plants (Figure 3C). Mature rosette leaves are always smaller in *tko-1* mutant plants than in wild type plants irrespective of plants' developmental stages (Figures 3A, **5A**). The size differences in the *atpgl3* single homozygous mutant plants and the *tko-1* triple mutant plants were not observed if the mutant plants were germinated and grown on agar plates.

**Figure 4:**
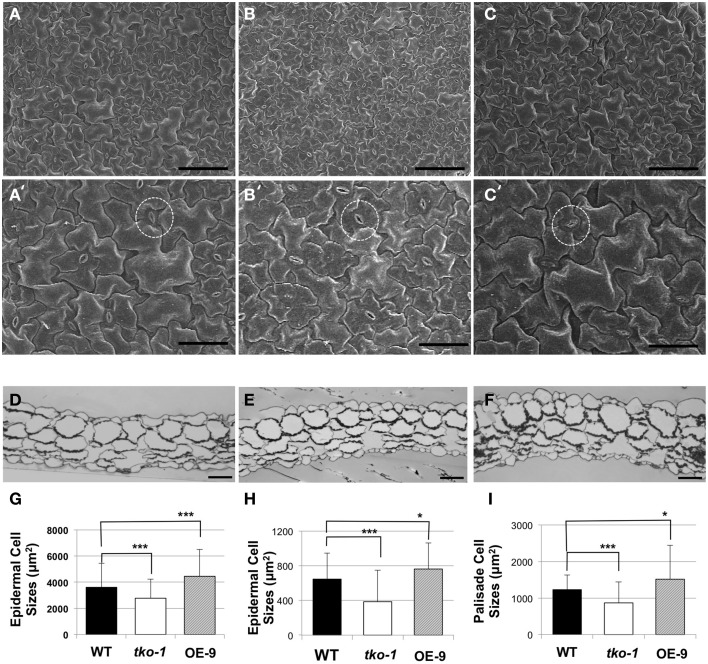
**Differences in cell sizes in leaves from 21-day-old plants. (A–C)** Adaxial epidermal cells under SEM (scale bar = 100 μm). **(A–C)** represent WT, *tko-1*, and OE-9, respectively. **(A'–C')** are higher magnification images of the samples in **(A–C)** (scale bar = 50 μm). Guard cells are marked with dashed circles. **(D–F)** Cross sections of the leaves, WT, *tko-1*, and OE-9, respectively (scale bar = 50 μm). **(G)** Adaxial epidermal cell sizes obtained from epidermal peels. (*n* = 135 epidermal cells from three leaves of each; WT, *tko-1*, and OE-9). **(H,I)** Sizes of adaxial epidermal cells and palisade cells from the cross sections of the leaves. Numbers of epidermal cells examined for averaging were 270, 175, and 200 for WT, *tko-1*, and OE-9, respectively. Numbers of palisade cells examined for averaging were 165, 150, and 255 for WT, *tko-1*, and OE-9, respectively. Error bars represent standard deviation. The significance of cell size differences between WT, *tko-1*, and OE-9 was determined by Student's *t*-test. ^*^P < 0.05, ^***^P < 0.001.

We imaged leaf epidermal cells by scanning electron microscopy (SEM) to determine why *tko-1* plants have smaller leaves. The epidermal cells in *tko-1* leaves are smaller (Figure 4A). We also measured surface areas of leaf epidermal cells by microscopic observation of epidermal peels. Surface areas of individual epidermal cells in wild type leaves averaged 3608 μm^2^ (*SD* = 1823 μm^2^), while epidermal cells of *tko-1* leaves had an average surface area of 2276 μm^2^ (*SD* = 1453 μm^2^) (Figures 4A,C,G). To compare cell sizes in the leaf parenchyma tissue, we prepared semi-thin sections of wild type and *tko-1* leaves and measured average areas of cross sections of epidermal cells and of palisade cells (Figures 4B, D, E). The *tko-1* leaves contained smaller epidermal and palisade cells than did those of the wild type (Figures 4B,H,I). These results were consistent with the leaf cell size differences observed in SEM samples after fracturing the leaves open (Figure S4).

A construct with *AtPGL3* cDNA along with its 1.7 kb native promoter was introduced into the triple mutant. The AtPGL3 construct rescued the phenotype of cell size reduction in the triple mutant (Figures 3A,B). Recently, a cotton protein (GhRDL1) homologous to the Arabidopsis RD22 BURP domain protein (AtRD22, At5g25610) was shown to promote cell elongation in the cotton fiber (Xu et al., 2013). However, when we compared homozygous T-DNA inserted mutant plants of *AtRD22* with wild type plants, the mutant plants did not show any sign of inhibited cell expansion (Figure S5).

### Overexpression of AtPGL3 promotes cell enlargement

To test whether *AtPGL3* is directly involved in cell growth, we produced transgenic lines in which *AtPGL3* is overexpressed by a CaMV 35S promoter. RT-PCR analysis indicated that transcript levels of *AtPGL3* are approximately three times higher in the transgenic plants than in wild type plants (Figure 3D). The high levels of *AtPGL3* resulted in plants with larger leaves and longer petioles than those of wild type plants (Figures 3A,B). When the cell sizes in epidermal layers were measured by epidermal peels, their average size was 4452 μm^2^ (*SD* = 2038 μm^2^), larger than the average epidermal cell size in wild type leaves (Figure 4C). The mesophyll cells beneath the epidermal layer were also observed in semi-thin sections to be enlarged relative to those in the wild type (Figures 4B,E,F). Even though activity of the *AtPGL3* promoter is strong in trichomes and in guard cells, these specialized epidermal cells in the *tko-1* triple mutant line or in the OE-9 overexpressor line did not exhibit any size differences relative to those in wild type leaves (Figure 4A).

### AtPGL3 is localized to the cell wall

**Figure 5:**
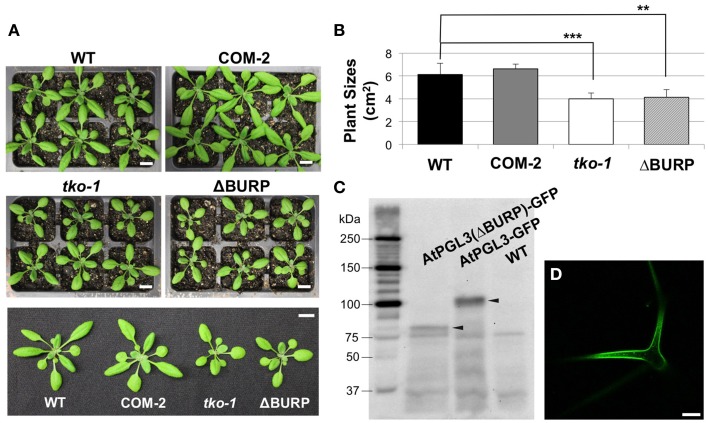
**Transformation of** ***tko-1*** **with AtPGL3-GFP and with AtPGL3(ΔBURP)-GFP (A)**. Twenty eight-day old wild type (WT), *tko-1* complemented with AtPGL3-GFP (COM-2), *tko-1* mutant, and *tko-1* mutant expressing AtPGL3-GFP lacking the BURP domain (ΔBURP). **(B)** Average plant sizes. Error bars indicate standard deviation. The double asterisk and the triple asterisk represent *P*-value < 0.01 and *P*-value < 0.005, respectively, when compared with WT by Student's *t*-test. **(C)** Immunoblot analysis of *tko-1* mutant lines expressing AtPGL3(ΔBURP)-GFP and AtPGL3-GFP. GFP proteins were visualized with the GFP antibody used for immunogold labeling in Figure 2. Arrowheads indicate GFP proteins of expected sizes. **(D)** GFP fluorescence from a leaf trichome of an AtPGL3(ΔBURP)-GFP plant. Scale bars in **(A)** = 1 cm, Scale bar in **(D)** = 50 μm.

To localize the AtPGL3 protein, we generated Arabidopsis plants expressing AtPGL3 fused with GFP at its C-terminus. The GFP fusion protein was expressed under the 1.7 kb native promoter and this GFP protein complemented phenotypes associated with inactivation of the in *tko-1* triple mutant plants (Figures 5A,B). AtPGL3-GFP fluorescence was detected in trichomes and in guard cells (Figures 2E–G), in agreement with the GUS promoter activity assay. GFP fluorescence was also observed in the leaf epidermal cells, where it overlapped with cell wall-specific fluorescence from propidium iodide (Figures 2H–J). To determine subcellular localization of AtPGL3-GFP, we performed immunogold labeling of the GFP transgenic line cells with an anti-GFP antibody. The immunogold particles were seen in the cell walls of trichome cells and of leaf epidermal cells (Figures 2K–M). The GFP-specific gold particles were scattered randomly over the epidermal cell wall (Figure 2N), suggesting that AtPGL3 is secreted into the apoplast.

### The BURP domain in AtPGL3-GFP is required for its complementation of tko-1

LePG1β in its mature form in ripening tomatoes does not contain the BURP domain, suggesting that the domain is cleaved when LePG1β is secreted to the cell wall (Zheng et al., 1992). To test whether the BURP domain of AtPGL3 is removed, we performed immunoblot analyses of protein extracts from *tko-1* mutant plants that were rescued by AtPGL3-GFP. A polypeptide of ~100 kDa was recognized by the GFP antibody from the triple mutant complemented by AtPGL3-GFP. The 100 kDa polypeptide matches the full length AtPGL3 protein (~70 kDa) combined with GFP (27 kDa), indicating that the functional GFP fusion protein contains the BURP domain. To test whether the BURP domain is needed for the GFP protein to rescue tko-1, we prepared a new construct in which the BURP domain of AtPGL3 was removed (from amino acid 397–626) and the truncated AtPGL3 (AtPGL3ΔBURP) was fused with GFP. The construct failed to restore *tko-1* back to the wild type size (Figures 5A,B), although we were able to detect the fusion protein by immunoblot analysis and by confocal microscopy imaging (Figure 5D). These results suggest that the BURP domain of AtPGL3 is critical for AtPGL3's role in cell growth.

### α-expansin6 is up-regulated in the AtPGL3 overexpressor line

Genes encoding proteins in a same pathway are often transcriptionally co-regulated (Schmid et al., 2005). To identify genes that are functionally related to *AtPGL3*, we searched the STRING v10 database that quantitatively predicts candidates for interacting partner proteins based on transcriptional correlations, published experimental results, and other parameters (http://string-db.org) (Szklarczyk et al., 2015).

**Figure 6:**
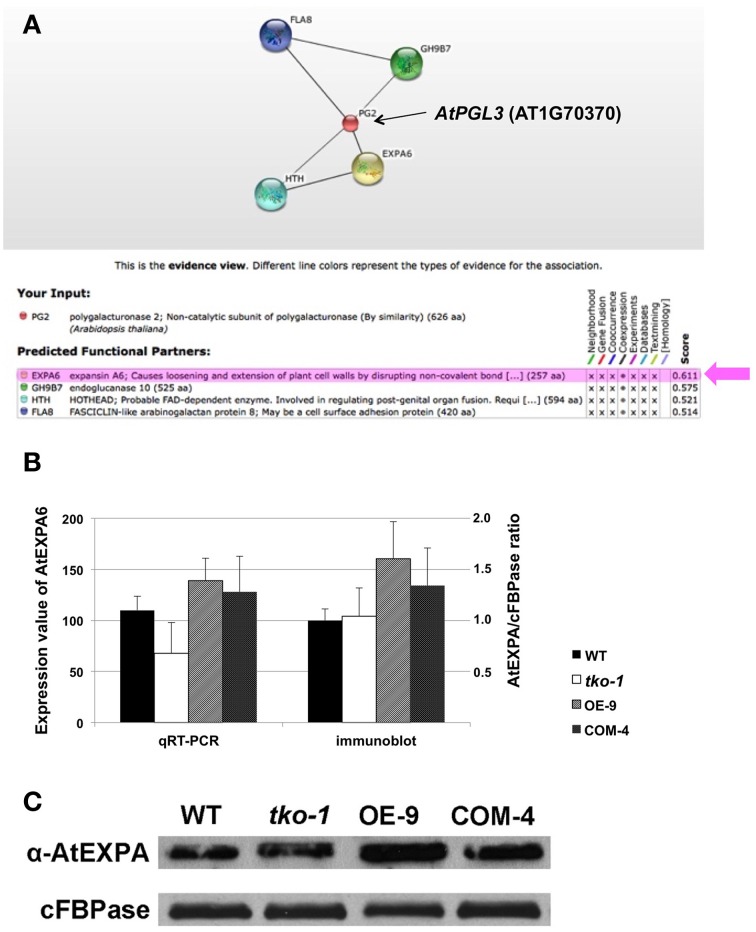
**qRT-PCR and immunoblot analyses of AtEXPA6 in the** ***AtPGL3*** **transgenic lines. (A)** A network view from the search of genes coexpressed with *AtPGL3* using the confidence threshold value of 0.5 from the STRING ver 10. PG2 (AT1G70370) in the network corresponds to *AtPGL3*. Beneath the network is a table showing the input gene (PG2), the prediction from coexpression datasets, and confidence scores. **(B)** Transcript levels of AtEXPA6 measured by qRT-PCR (left y-axis) and levels of α-expansins estimated by immunoblot analysis (right y-axis). Rosette leaf samples from 21-day-old wild type (WT), triple mutant (*tko-1*) and AtPGL3 overexpressor (OE-9) plants were examined. Amounts of α-expansins were approximated by polypeptide intensities in the blots and normalized with amounts of cytosolic fructose -1,6-bisphosphatase (cFBPase) in the same samples. qRT-PCR and immunoblot analyses were performed with samples from six different plants. **(C)** A representative immunoblot of the three genotypes with the anti-α-expansin10 antibody.

Because we are interested in genes whose expression is linked to *AtPGL3* expression, the prediction was made exclusively based on evidence of coexpression (Figure 6A). *AtEXPA6* (α-expansin6, At2G28950) had the highest confidence score of 0.611, followed by AtGH9B7 (endoglucanase, At1G75680) that had a confidence score of 0.575. AtEXPA6 is an α-expansin that might be functionally associated with the cell enlargement effect of AtPGL3. Therefore, we examined expression levels of *AtEXPA6* in smaller and larger rosette leaf samples from the *tko-1* triple mutant and from the *AtPGL3* overexpressor line plants by qRT-PCR and immunoblot analyses (Figures 6B,C). Arabidopsis has 26 α-expansins (Lee et al., 2001) and AtEXPA6 is closely related to α-AtEXPA10 (At1g26770). They share 68.7% amino acid sequence identity. Because AtEXPA6 specific antibody is not available, we utilized an antibody generated with AtEXPA10 to estimate protein levels of AtEXPA6. Transcription levels of *AtEXPA6* are reduced in the triple mutant samples but are increased in the samples from *AtPGL3* overexpressor plants, suggesting that AtEXPA6 is involved in the cell size differences in the two transgenic lines (Figure 6B). In the immunoblot analyses using the anti-α-expansin10 antibody, the amount of α-expansin polypeptides is increased in the enlarged leaves of AtPGL3 overexpressor plants (Figures 6B,C). However, the triple mutant leaves have as much α-expansin polypeptides as the WT leaves, probably because the anti-AtEXPA10 antibody recognizes α-expansins other than AtEXPA6.

## Discussion

The regulation of cell size is an important research topic for understanding plant development, as well as for plant bioengineering. Morphogenesis and biomass production in plants are dependent on cell division and cell expansion, but manipulation of cell expansion is more practical for bioengineering because defects in cell division often lead to embryo/seedling lethality (Lukowitz et al., 1996; Kang et al., 2003; Brukhin et al., 2005). In this paper, we demonstrated that AtPGL3 is an Arabidopsis BURP domain protein secreted to the cell wall, and that its expression level is correlated with the expansion of leaf epidermal cells and of leaf ground tissue cells.

As demonstrated by Xiao et al. (2014), cell expansion involves the breakdown of pectin polysaccharides. AtPGLs are related to the LePG1β subunit of the polygalacturonase complex that decomposes pectin polysaccharides in ripening tomato fruits, but we did not find strong evidence that AtPGL3 and polygalacturonases are co-expressed in Arabidopsis. Another difference between PG1β and AtPGL3 is that the AtPGL3 protein does not lose its BURP domain during its maturation. The mature form of LePG1β consists mostly of the central FXXY domain after the BURP domain at the C-terminus and the short segment in the N-terminus are cleaved (Figure 1A) (Zheng et al., 1992). By contrast, the BURP domain of AtPGL3 is indispensable for rescuing the *tko-1* phenotype. Therefore, AtPGL3's role in cell expansion in rosette leaves is likely to operate in a mode distinct from that of LePG1β for softening tomato fruits.

Defective cell wall assembly, such as aberrant cellulose biosynthesis (Fagard et al., 2000) or inhibition of non-cellulosic polysaccharide secretion (Kang et al., 2011), leads to reduced cell expansion. By contrast, loosening of the cell wall by increasing the expression of an Arabidopsis expansin promoted cell expansion (Cho and Cosgrove, 2000). Transcript levels of *AtPGL3* are correlated with those of *AtEXPA6* in the transgenic lines where the expression of *AtPGLs* has been altered. An increase in cell sizes by *AtPGL3* overexpression correlates with an increased amount of α-expansins. These results suggest that the effects of AtPGL on cell size determination occur through the cell wall loosening effects of α-expansins.

AtPGL3 is not the first protein that has been shown to promote cell expansion in association with expansins. In a recent study of a cotton protein, GhRDL1, it was shown that overexpression of *GhRDL1* increased cotton fiber length and seed cell sizes (Xu et al., 2013). Because GhRDL1 interacts with a cotton expansin and its growth promoting effect is synergistically enhanced when it is co-expressed with the expansin in cotton plants, it was suggested that increased cotton fiber yield in the *GhRDL1* overexpressor lines is mediated through the expansin. GhRDL1 is a BURP domain protein and its closest Arabidopsis homolog is AtRD22 (Xu et al., 2013). We examined T-DNA inserted mutants of *AtRD22* but did not observe a cell size reduction in the mutant plants. Arabidopsis leaves express more AtRD22 when they are under moisture stress and a loss-of-function allele of AtRD22 exhibited delayed senescence after drought stress (Harshavardhan et al., 2014). These findings suggest that the biological functions of AtRD22 are more likely related to stress responses than to cell size regulation. Similarly, a soybean homolog of AtRD22, GmRD22, is involved in lignin biosynthesis to fortify the cell wall in response to osmotic stresses (Wang et al., 2012).

Although more research should be performed to characterize the functional association between expansins and AtPGLs, correlative expression of AtEXPA6 and AtPGL3, which occurs at the transcript level as well as at the protein level (Figures 6B,C), agrees with the notion that expression levels of physically interacting proteins are linked (Ge et al., 2001). It has been suggested that genes encoding subunits of a protein complex evolve to be regulated together because non-coordinated expression of a subunit in a protein complex could pose detrimental effects (Papp et al., 2003; Fraser et al., 2004). For example, proteosomes are made up of many subunits and null mutant alleles of one of its subunits altered the expression levels of the other subunits (Lee et al., 2012). It is also possible that AtPGL may serve as a chaperone for expansins through the secretory pathway so that they are deposited to the cell wall properly. This would explain the increased amount of expansins in the AtPGL3 overexpressor lines.

T-DNA mutant plants of *AtPGL1* and *2* exhibited no distinct phenotypes when compared with wild type plants. *atpgl3* mutant plants were slightly smaller than wild type plants, although *AtPGL3* is the most highly transcribed gene among the three PG1β –like protein of Arabidopsis. But disruption of *AtPGL1* and *AtPGL2* in the *atpgl3* mutant plants augmented the effect of *AtPGL3* inactivation (Figure 3B). These data suggest that AtPGL3 can compensate for inactivation of other *AtPGLs* and that *AtPGL1* and *2* are also involved in the cell expansion pathway that *AtPGL3* contributes to.

## Materials and methods

### Plant materials and growth conditions

*Arabidopsis thaliana* (ecotype Columbia-0) seeds were vapor-phase sterilized as described in Clough and Bent (1998) and soaked in water at 4°C for 2 days. They were sowed on soil and grown under a 16 h light/8 h dark cycle at 22°C.

T-DNA inserted mutant seeds of *atpgl1, atpgl2*, and *atpgl3* were obtained from ABRC (Arabidopsis Biological Resource Center, Columbus, OH). Primers for genotyping T-DNA inserted mutant plants were designed as suggested by SIGnAL (http://signal.salk.edu/tdnaprimers.2.html). Transformants were screened by growing them on 0.8% agar medium containing 0.5X Murashige and Skoog salts and 1% sucrose, with 50 μg/ml hygromycin or kanamycin.

### Amino acid sequence alignment, RT-PCR, and qRT-PCR

The amino acid sequences of the three AtPGLs were aligned and their homology was calculated with ClustalW as described in Xiong et al. (2014). The conserved domain was mapped using the UniProt database (www.uniprot.org). Total RNA samples were isolated from 14-day-old seedlings with a total RNA purification kit (Norgen Biotek, Ontario, Canada) and complementary DNA (cDNA) was synthesized with SuperScript III First-Strand Synthesis System (Invitrogen, CA). The cDNA was amplified using the primer sets listed in Table S1 (AtPGL L1+R1, L2+R2, L3+R3, and AtPGL3 RT F+R). Total RNA samples were isolated from 6-day-old seedlings (cotyledons), 14-day-old seedlings (mature rosette leaves, inflorescence stems), and mature flowers. Their cDNA samples were prepared as described above. Quantitative reverse transcription-PCR (qRT-PCR) was performed with SYBR Green (Agilent Technologies, CA) for *AtPGL1, AtPGL2*, and *AtPGL3* genes in the cDNA from the five different tissues. *AtEXPA6* genes were amplified from cDNA from rosette leaves from 21-day-old plants. Primer sets for qRT-PCR are listed in Table S1. The expression levels of the target genes were measured from the qRT-PCR results as described in Xiong et al. (2011). qRT-PCR analyses were performed three times with leaf samples from different plants.

### GUS assay of promoter activity

Approximately 1700 base pairs (bp) of the AtPGL3 promoter were amplified from genomic DNA from mature leaves with the primer set listed in Table S1 (pAtPGL3 BamHI F + pAtPGL3 NcoI R). The amplified promoter DNA was inserted into the pCAMBIA1305 binary vector in frame with its *GUS* gene using the BamHI and NcoI restriction enzyme sites.

The cloned construct was introduced into *Arabidopsis* plants by the floral dip *Agrobacterium*-mediated transformation method (Clough and Bent, 1998). The seeds from the transformed *Arabidopsis* plants were harvested and transgenic plants were grown as described above.

Eighteen-day-old transgenic seedlings were stained with 5-bromo-4-chloro-3-indolyl-β-D-glucuronide (1 mM) in 50 mM phosphate buffer (pH 7.2) containing potassium ferrocyanide (5 mM), potassium ferricyanide (5 mM), and Triton X-100 (0.2%, v/v) for 24 h at 37°C (Kang et al., 2003). Stained seedlings were bleached with 70% ethanol for 48 h at room temperature. The floral organs and siliques were dissected out from the transgenic plants and stained as described above. The stained tissue samples were observed under an EMZ dissecting microscope (Meiji Techno America, CA) or an Olympus BH2 compound light microscope (Olympus America, PA).

### Generating transgenic lines expressing AtPGL3-GFP fusion proteins and fluorescence microscopy imaging

The *AtPGL3* gene and ~1700 bp of its promoter were amplified and subcloned into a pCAMBIA1302 vector using SalI and NcoI restriction sites with primers listed in Table S1 (pAtPGL3 SalI F + AtPGL3 NcoI R). Using the restriction sites, the cDNA was ligated in the 5′ end of the GFP coding region of pCAMBIA1302. According to the prediction by Uniprot, AtPGL3 cDNA lacking the BURP domain was amplified with primers, pAtPGL3 SalI F and AtPGL3(ΔBURP) R, and ligated into the pCAMBIA1302 vector. The resulting construct was transformed into *Arabidopsis* plants and transformants were selected. The GFP expression in the transgenic plants was confirmed by immunoblot analysis using an antibody against GFP (Cat No. sc-8334, Santa Cruz Biotechnology, CA). GFP fluorescence from 18-day-old seedlings was observed on a stereo microscope equipped with fluorescent illumination (Leica Microsystems, IL) or on an LSM 5 PASCAL confocal microscope (Carl Zeiss Microscopy, Germany). To colocalize AtPGL3-GFP with the cell wall, we stained leaf samples with propidium iodide (100 μg/ml) for 30 s and rinsed with distilled water. The stained cell wall was visualized with a HeNe laser (543 nm excitation) and a longpass detection filter (560 nm). The images of the propidium iodide and GFP channels were electronically merged with Photoshop (Adobe Systems, CA).

### Preparation of the AtPGL3 overexpressor and complementation lines

The *AtPGL3* cDNA was amplified with AtPGL3 XmaI F and AtPGL3 SacI R (Table S1) and the amplified fragment was cloned into XmaI and SacI restriction sites of a pBI121 binary vector carrying a CaMV 35S promoter. Expression levels of *AtPGL3* in the transformants were compared to those of wild type plants by RT-PCR.

The *AtPGL3-GFP* construct was used for the complementation test. The construct was transformed into *tko-1 Arabidopsis* mutants via *Agrobacterium*, and transformants were screened. The presence of the complementation construct and expression of the *AtPGL* genes were verified by RT-PCR as described above.

### Plant size measurement

Wild type (WT), the triple mutant (*tko-1*), an overexpressor line (OE-9), and a complemented line (COM-4) were sowed and grown in soil. 21-day-old plants of each line were photographed and their outlines were drawn in ImageJ to calculate their sizes (Image Processing and Analysis in Java, ver. 2.0). For the BURP domain-truncated lines transformed into *tko-1*, 21-day-old plants were grown and measured as described above along with wild type, *tko-1*, and complementation lines. For the *atrd22* mutant, 28-day-old WT and mutant plants were compared.

### Cell size measurement

To compare the epidermal layers, the fourth rosette leaves were anchored on microscope slides by double sided tape. The other layers of cells were removed by scraping with forceps and were then washed away with water. The epidermal cell layers stuck to the slide were stained with toluidine blue and rinsed immediately with water.

To measure mesophyll cell sizes, fourth rosette leaves were cut into small pieces that were placed in fixative [4% (v/v) paraformaldehyde, 2% (v/v) glutaraldehyde in 0.1 M cacodylate buffer] and were held overnight at 4°C. The leaves were rinsed with 0.1 M cacodylate buffer several times and secondarily fixed in 1% osmium tetroxide in 0.1 M cacodylate buffer overnight at 4°C, followed by several water washes. The leaves were subsequently dehydrated through a graduated series of ethanol (13–100%) and infiltrated with a graduated series of LR White resin (25–100%). The samples were embedded in 100% LR White and were cured at 60°C for 2 days (Koh et al., 2012). The leaves in resin were semi-thin sectioned (500 nm) using an Ultracut UCT ultramicrotome (Leica Microsystems, IL). The sections were placed on slides and were stained with Toluidine Blue. Epidermal peels and LR White sections were imaged with an Olympus BH2 compound light microscope (Olympus America, PA). Cell sizes were calculated with ImageJ from cell outlines in micrographs. The *P*-values were calculated with the T.TEST function (Student's *t*-Test) in the Excel software (Microsoft, WA). Tails and types for the T.TEST function were set to two-tailed distribution and homoscedastic test, respectively.

### Scanning electron microscopy (SEM), transmission electron microscopy (TEM), and immunogold labeling

For SEM, rosette leaf samples were collected using biopsy punches (2 mm diameter). They were fixed with 4% (v/v) paraformaldehyde, 2% (v/v) glutaraldehyde in 0.1 M cacodylate buffer. Dehydration, critical point drying, scanning electron microscopy imaging, and image analyses were performed as described in Koh et al. (2012). For immunogold labeling and TEM imaging, rosette leaves from wild type and complemented lines were collected and fixed as described above. They were secondarily fixed in 1% (v/v) aqueous uranyl acetate overnight at 4°C. After rinsing with water several times, they were dehydrated and infiltrated with LR White resin. Rosette leaves were also processed by high-pressure freezing. The frozen samples were freeze substituted and embedded in HM20 resin (Electron Microscopy Sciences, PA) according to Donohoe et al. (2013). The samples were thin sectioned (70 nm) using an Ultracut UCT ultramicrotome (Leica Microsystems, IL). The sections were picked up on formvar coated nickel slot grids and immunogold labeling was carried out as described in Kang (2010).

### Immunoblot analysis

Protein extraction and immunoblot analyses were performed as previously described in Lee et al. (2013). Immunoblots were probed with an anti-GFP antibody (Sc9996, Santa Cruz Biotechnology, CA; 1:3000 dilution) and an anti-AtEXPA10 antibody (ABIN678788, Bioss Antibodies, MA; 1:1000 dilution). Polypeptide density measurements used for preparing the histogram of α-expansin/cytosolic fructose 1,6 bisphosphatase ratios were made with ImageJ.

### Conflict of interest statement

The authors declare that the research was conducted in the absence of any commercial or financial relationships that could be construed as a potential conflict of interest.

## Supplementary material

Figure S1 **Amino acid alignment of AtPGL1, AtPGL2, AtPGL3 and LePG1β using the CLASTALW multiple alignment tool**. Identity (^*^), strong similarity (**:**), and weak similarity (.) among the four proteins are denoted beneath the LePG1β sequence. The FXXY repeats are highlighted in light gray. The conserved residues in the BURP domain are in black boxes.

Figure S2 **Amino acid alignment of AtPGL1, AtPGL2, and AtPGL3 using the CLUSTALW multiple alignment tool**. The asterisks, colons, and dots under the AtPGL2 sequence denote identical, strongly similar, and weakly similar residues, respectively. The 21 FXXY repeats are highlighted in light gray. The conserved residues in the BURP domain are in black boxes.

Figure S3 **Relative expression levels of** ***AtPGL*** **transcripts at different developmental stages**. The expression values were acquired from a 22 K microarray of the Genevestigator plant biology database (https://genevestigator.com/gv/).

Figure S4 **(A–C)** Cross sectional views of 21-day-old plant leaves imaged by SEM. **(A)** WT; **(B)** *tko-1*; and **(C)** OE-9 (scale bar: 50 μm). **(D)** Average cell sizes in the SEM micrographs. Error bars represent standard deviation. The triple asterisk indicates a *P*-value < 0.001. Electron micrograph of a wild type (WT) leaf section after immunogold labeling with the anti-GFP antibody used for localizing AtPGL3-GFP in Figure 2E. No gold particles are detected in the WT section. Scale bar = 1 μm.

Figure S5 **Size comparison of 28-day-old WT and** ***atrd22*** **mutant plants. (A)** Twenty eight-day-old WT and *atrd22* mutant plants. Scale bar = 1 cm. **(B)** Average sizes of 28-day-old WT and *atrd22* mutant plants. Error bars indicate standard deviation.
