# Supplementary figures and images for: AtPGL3 is an Arabidopsis BURP domain protein that is localized to the cell wall and promotes cell enlargement
Source: Front Plant Sci. 2015 Jun 9;6:412. doi: 10.3389/fpls.2015.00412 (PMC4460304; doi:10.3389/fpls.2015.00412)

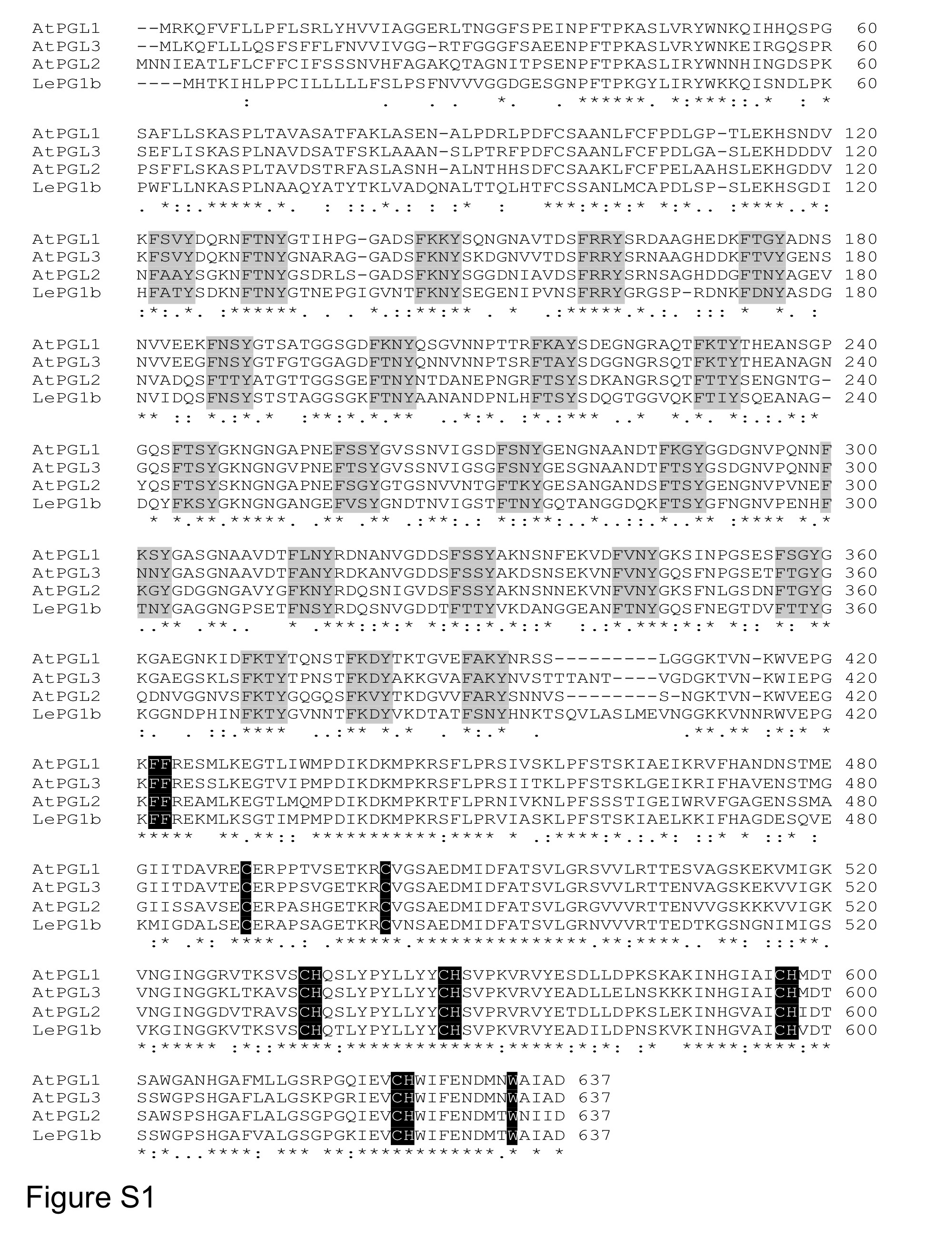

Supplement: Figure S1 — Amino acid alignment of AtPGL1, AtPGL2, AtPGL3 and LePG1β using the CLASTALW multiple alignment tool. Identity (*), strong similarity (:), and weak similarity (.) among the four proteins are denoted beneath the LePG1β sequence. The FXXY repeats are highlighted in light gray. The conserved residues in the BURP domain are in black boxes. [file Image1.JPEG]

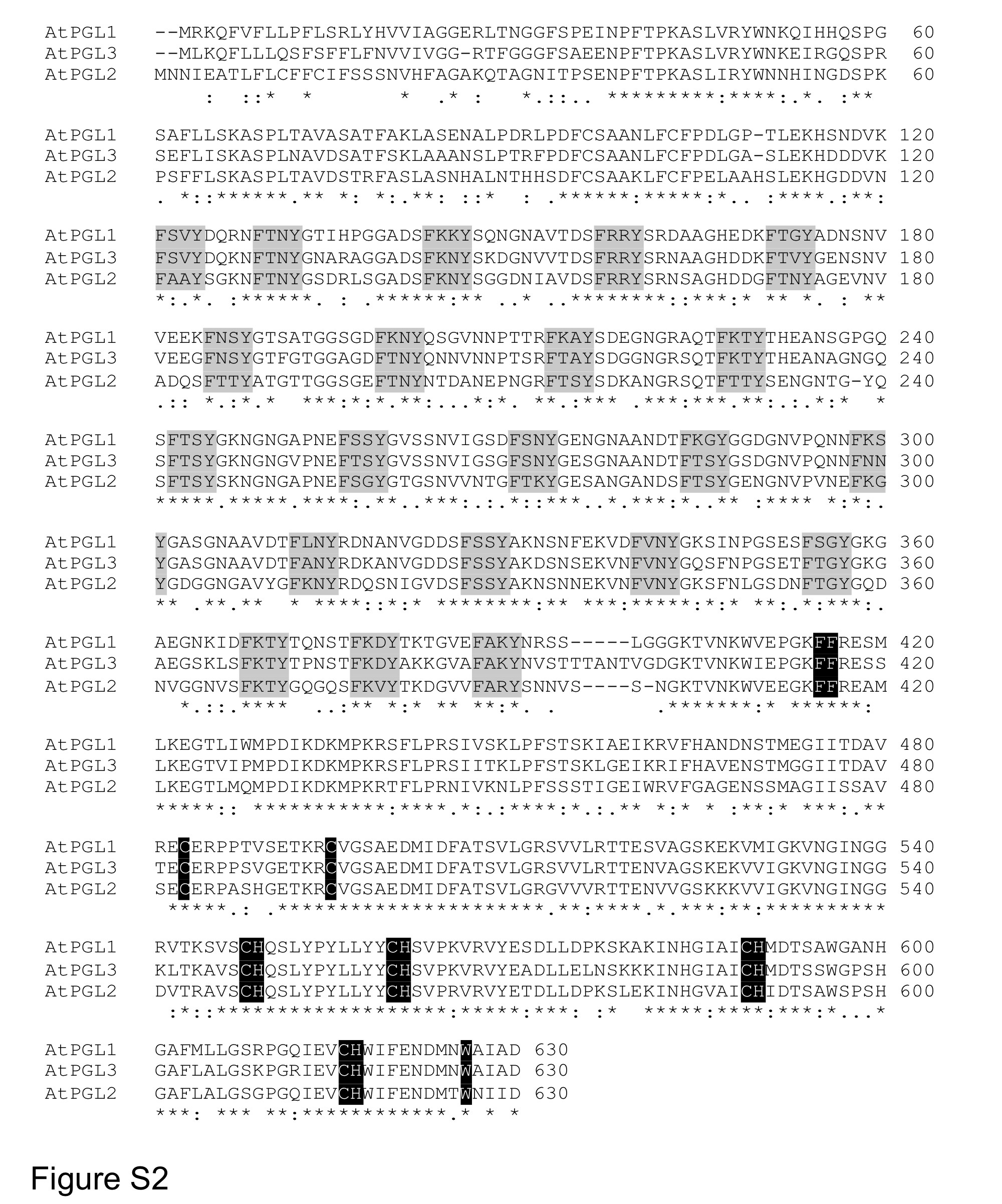

Supplement: Figure S2 — Amino acid alignment of AtPGL1, AtPGL2, and AtPGL3 using the CLUSTALW multiple alignment tool. The asterisks, colons, and dots under the AtPGL2 sequence denote identical, strongly similar, and weakly similar residues, respectively. The 21 FXXY repeats are highlighted in light gray. The conserved residues in the BURP domain are in black boxes. [file Image2.JPEG]

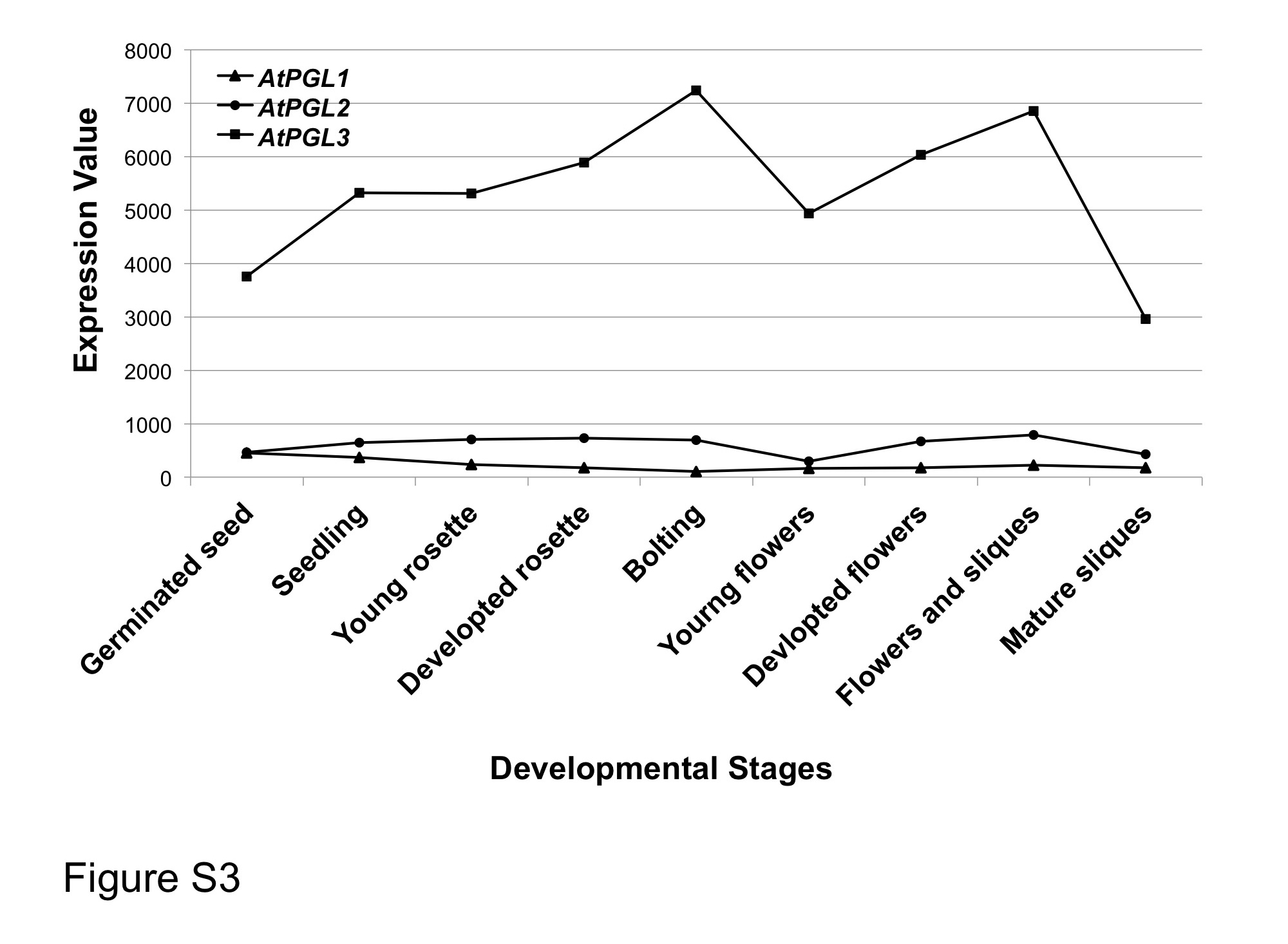

Supplement: Figure S3 — Relative expression levels of AtPGL transcripts at different developmental stages. The expression values were acquired from a 22 K microarray of the Genevestigator plant biology database (https://genevestigator.com/gv/). [file Image3.JPEG]

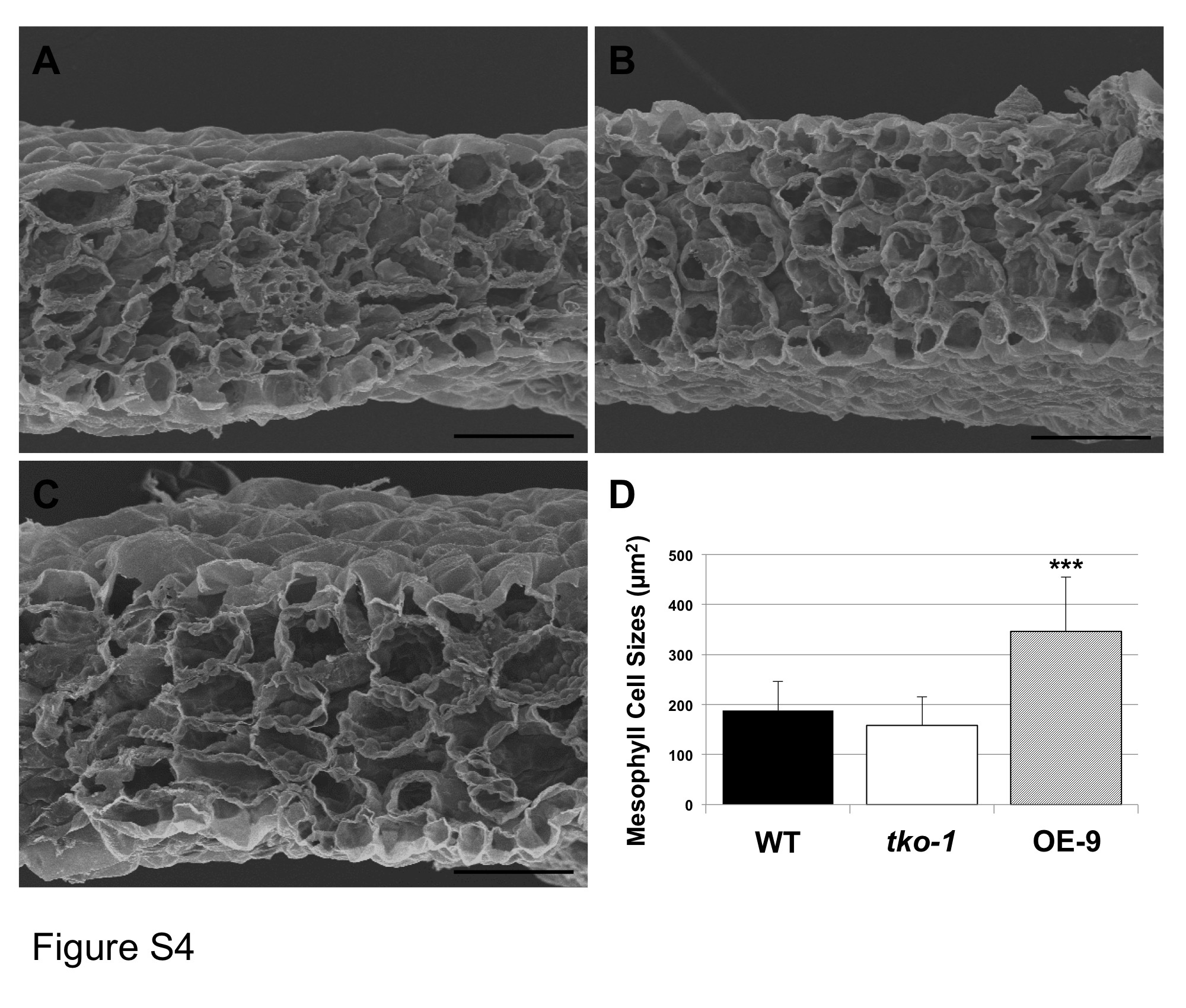

Supplement: Figure S4 — (A–C) Cross sectional views of 21-day-old plant leaves imaged by SEM. (A) WT; (B) tko-1; and (C) OE-9 (scale bar: 50 μm). (D) Average cell sizes in the SEM micrographs. Error bars represent standard deviation. The triple asterisk indicates a P-value < 0.001. Electron micrograph of a wild type (WT) leaf section after immunogold labeling with the anti-GFP antibody used for localizing AtPGL3-GFP in Figure 2E. No gold particles are detected in the WT section. Scale bar = 1 μm. [file Image4.JPEG]

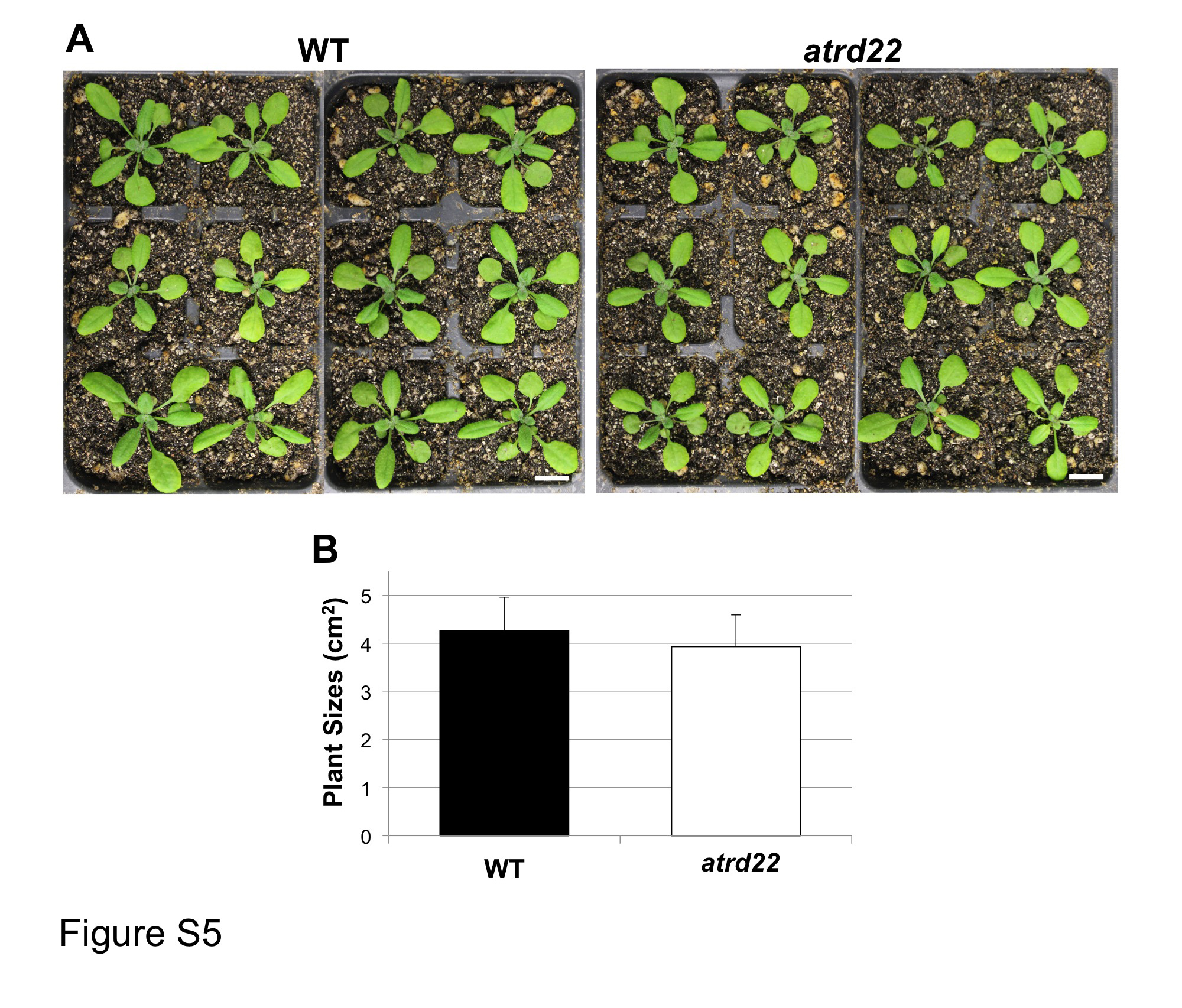

Supplement: Figure S5 — Size comparison of 28-day-old WT and atrd22 mutant plants. (A) Twenty eight-day-old WT and atrd22 mutant plants. Scale bar = 1 cm. (B) Average sizes of 28-day-old WT and atrd22 mutant plants. Error bars indicate standard deviation. [file Image5.JPEG]
