# Supplementary material for: AtPGL3 is an Arabidopsis BURP domain protein that is localized to the cell wall and promotes cell enlargement
Source: Front Plant Sci. 2015 Jun 9;6:412. doi: 10.3389/fpls.2015.00412 (PMC4460304; doi:10.3389/fpls.2015.00412)
Supplement: Supplementary file 6 [file Table1.DOCX]

| **Supplemental Table S1. List of Primers** | |
| --- | --- |
| Primers | Sequences |
| AtPGL1 L1 | 5’- CTCGTCGTATGCCAAGAACTC -3’ |
| AtPGL1 R1 | 5’- TCCAATGGCATACTTCGATCT -3’ |
| AtPGL2 L2 | 5’- TTGACTTTTCCAATGACGACC -3’ |
| AtPGL2 R2 | 5’- GTCTGCGTCTTCGATGAAGAG -3’ |
| AtPGL3 L3 | 5’- CTGGAACAAAGAGATTCGTGG -3’ |
| AtPGL3 R3 | 5’- TTATTTACGGTTTTGCCATCG -3’ |
| ACT2 F | 5’- GTCGTATGCCAAGAACTCAAAC -3’ |
| ACT2 R | 5’- CCCTCAGCTCCTTTACCATAAC -3’ |
| AtPGL3 RT F | 5’- GGCAATGCTCGTGCCGGAGGA-3’ |
| AtPGL3 RT R | 5’- CGCGGTGGTTGTTGAAACGTTGTA -3’ |
| AtPGL1 qRT F | 5’- CCTATACTCACGAAGCCAACTC -3’ |
| AtPGL1 qRT R | 5’- GGAGACACCGTAAGAAGAGAAC -3’ |
| AtPGL2 qRT F | 5’- CGGCAAGAACTTCACAAACTAC -3’ |
| AtPGL2 qRT R | 5’- CTGTAACGTCGGAATGAGTCTAC -3’ |
| AtPGL3 qRT F | 5’- CTACGGAAGCGACGGTAATG -3’ |
| AtPGL3 qRT R | 5’- CGTTGGCCTTGTCTCTGTAA -3’ |
| pAtPGL3 BamHI F | 5’- GGATCCACACGACAATGTTAAAATGTTG -3’ |
| pAtPGL3 NcoI R | 5’- CCATGGTTGTTCTTCTTCTAGTGTCTA -3’ |
| pAtPGL3 SalI F | 5’- GTCGACCGCCATTGATTTTCATGC -3’ |
| AtPGL3 NcoI R | 5’- CCATGGTATCAGCGATAGCCCAGTTC -3’ |
| AtPGL3(ΔBURP) R | 5’- CCATGGAGACGGTGTTCGCGGTGGTT-3’ |
| AtPGL3 XmaI F | 5’- CCCGGGATGCTCAAACAGTTTCTTCTT-3’ |
| AtPGL3 SacI R | 5’- GAGCTCCTAATCAGCGATAGCCCAGTTC -3’ |
| AtEXPA6 qRT F | 5’- GGAACACATACTTCGTGGATGA -3’ |
| AtEXPA6 qRT R | 5’- GGTCACTGCTTGTGACTCTAAA -3’ |
